# Commentary: Effects of Sleep on Word Pair Memory in Children–Separating Item and Source Memory Aspects

**DOI:** 10.3389/fpsyg.2018.01022

**Published:** 2018-06-19

**Authors:** Jennifer M. Johnson, Simon J. Durrant

**Affiliations:** School of Psychology, University of Lincoln, Lincoln, United Kingdom

**Keywords:** declarative memory consolidation, sleep, children, hippocampus, prefrontal cortex

Evidence of sleep-dependent memory consolidation in adults has become widespread in the last two decades (Rasch and Born, 2013). However, investigations of similar consolidation in children are still at an early stage (Maski et al., 2015).

Previous research has shown sleep-dependent memory consolidation in children on tasks such as lexical integration of novel words (Henderson et al., 2012) and spatial location learning (Kurdziel et al., 2013). However, this appears to be limited to declarative memory tasks; procedural learning tasks do not show the same sleep-related benefit in children that is seen in adults (Fischer et al., 2007; Wilhelm et al., 2008). Declarative memory tasks are strongly hippocampal-dependent (Squire, 1992), and consolidation of hippocampal memories is particularly associated with slow wave sleep (SWS) (Diekelmann et al., 2009; Born and Wilhelm, 2012). It has therefore been suggested that the large amount of SWS seen during childhood (Ohayon et al., 2004) is the reason declarative memories show sleep-dependent consolidation in children.

However, not all declarative memories are equal. In particular, a key distinction can be drawn between the memory of an object (item memory) and the context in which it was encountered (source memory). The recent paper by Wang et al. (2017) used a clever design to separate these two forms of memory, and examined their consolidation in children across an 11 h interval containing wake or sleep or a shorter 1 h wake interval, and compared this to adults with an 11 h sleep or 1 h wake interval. The design used two lists of word pairs and the innovation was to measure memory not only for the target words using cued recall (item memory), but also for memory of which list they came from (source memory). When comparing adults to children, a key finding was that memory for word pairs was lower after sleep in children (compared to before), but showed no reduction after sleep in adults (Figure 2B in the original paper). Interestingly, this turned out to be driven entirely by the word pairs without source memory; where the item and source had both been remembered, no difference was seen.

The authors interpreted their findings in the context of less distinct encoding in children leading to more rapid unbinding of items and sources. However, this does not explain why item-only memory declines across sleep in children but item-source memory remains intact, and why the opposite pattern (stronger consolidation across sleep of item-only memory compared to item-source memory) is seen in adults.

We believe that the influential model by Preston and Eichenbaum (2013) allows a more comprehensive framework in which to explain the findings. This model postulates the existence of a “what” stream of cortical processing from the dorsal hippocampus through the perirhinal cortex, and a “where” stream from the dorsal hippocampus through the parahippocampal cortex. Additionally, the ventral hippocampus connects to the medial prefrontal cortex (mPFC), where pre-existing schemata influence subsequent memory processing (Tse et al., 2007; van Kesteren et al., 2010, 2012), and exert a top-down influence on “what” facilitating segregation from “where.” Importantly, Eichenbaum (2014, 2017) has suggested that the “where” stream also processes temporal context information in a similar way, i.e., it may also operate as a “when” stream.

A key difference in the declarative memory systems of children and adults is that the density of hippocampal synapses in children reaches similar levels to adults within the first 6 months of life (Seress and Ábrahám, 2008), but the frontal lobes have a prolonged developmental course (Luna and Sweeney, 2004) with myelination (Giedd et al., 1999), synaptic density counts (Huttenlocher, 1990) and overall function of the prefrontal cortex not reaching adult levels until late adolescence (Huber and Born, 2014). In children we should therefore expect to see stronger bottom-up processing, and a reduced top-down influence of existing schemata, which would otherwise typically strengthen item (“what”) memory at the expense of list context (“where/when”). This is supported by previous evidence confirming that more rapid consolidation of schematic memories occurs during sleep and involves the mPFC (Durrant et al., 2015), and that sleep-dependent consolidation leads to a reduction in associated contextual information (Cairney et al., 2011).

For the Wang et al. (2017) study, greater decontextualization and consolidation during sleep in adults compared to children would show up as a bigger reduction in item-only memory in children and this is exactly what the authors found (Figure 2D). However, we would expect to see relatively little change across sleep for items with their original contexts intact in both children and adults, reflecting equal hippocampal development, and we see this too (Figure 2C). This argument is further strengthened by the fact that the relationship between SWS and memory consolidation seen in the study in children was limited to items which retained their context. It also explains why the children showed little sleep-dependent benefit for items which have lost their contextual cues (Figure 1F).

In children, memory for the lists themselves was also stronger after sleep compared to wake (Figure 1G), while in adults, no significant enhancement of list memory was seen (Supplementary Material). If memory for the lists makes use of the contextual stream, the absence of strong mPFC influence allows both streams to consolidate during sleep, whereas the top-down mPFC influence in adults strengthens the “what” stream at the expense of the “where/when” stream (hence list memory is not enhanced after sleep compared to wake in adults).

The neural basis of our interpretation could be tested in fMRI, where we predict that prefrontal activation will be stronger in adults during sleep and associated with item-only memory, while hippocampal activation would be associated with both item and source memory, but more strongly for items which retain their context.

The Wang et al. (2017) paper has shown the clear benefits of measuring different aspects of declarative memory in a single study. We hope this new interpretation will help further improve our understanding of different memory systems and stimulate research in this area.

## Author contributions

All authors listed have made a substantial, direct and intellectual contribution to the work, and approved it for publication.

### Conflict of interest statement

The authors declare that the research was conducted in the absence of any commercial or financial relationships that could be construed as a potential conflict of interest.
